# MiR-15b and miR-322 inhibit *SETD3* expression to repress muscle cell differentiation

**DOI:** 10.1038/s41419-019-1432-5

**Published:** 2019-02-22

**Authors:** Meng-Jie Zhao, Jun Xie, Wen-Jie Shu, Hong-Yan Wang, Jianping Bi, Wei Jiang, Hai-Ning Du

**Affiliations:** 1 Hubei Key Laboratory of Cell Homeostasis, College of Life Sciences, Wuhan University, 430072 Wuhan, China; 2 Hubei Key Laboratory of Medical Information Analysis & Tumor Diagnosis and Treatment, 430074 Wuhan, China; 3 Medical Research Institute, School of Medicine, Wuhan University, 430071 Wuhan, China

## Abstract

SETD3 is a member of SET-domain containing methyltransferase family, which plays critical roles in various biological events. It has been shown that SETD3 could regulate the transcription of myogenic regulatory genes in C2C12 differentiation and promote myoblast determination. However, how SETD3 is regulated during myoblast differentiation is still unknown. Here, we report that two important microRNAs (miRNAs) could repress *SETD3* and negatively contribute to myoblast differentiation. Using microRNA (miRNA) prediction engines, we identify and characterize miR-15b and miR-322 as the primary miRNAs that repress the expression of *SETD3* through directly targeting the 3’-untranslated region of *SETD3* gene. Functionally, overexpression of miR-15b or miR-322 leads to the repression of endogenous *SETD3* expression and the inhibition of myoblast differentiation, whereas inhibition of miR-15b or miR-322 derepresses endogenous *SETD3* expression and facilitates myoblast differentiation. In addition, knockdown *SETD3* in miR-15b or miR-322 repressed myoblasts is able to rescue the facilitated differentiation phenotype. More interestingly, we revealed that transcription factor E2F1 or FAM3B positively or negatively regulates miR-15b or miR-322 expression, respectively, during muscle cell differentiation, which in turn affects *SETD3* expression. Therefore, our results establish two parallel cascade regulatory pathways, in which transcription factors regulate microRNAs fates, thereby controlling *SETD3* expression and eventually determining skeletal muscle differentiation.

## Introduction

Skeletal muscle differentiation is a complex process orchestrated by a family of myogenic regulatory factors (MRFs), including MyoD, myogenin, MRF4, and Myf5^1,2^. Expression of MyoD and Myf5 in the initial stages of differentiation induces expression of myogenin and muscle-specific transcription factors MEF2, whereas myogenin and MRF4 are expressed in the late stages of differentiation to activate the myogenic program by induction of muscle gene expression and silence of cell cycle-related gene expression^2–4^. Moreover, the functional interplay between key myogenic transcriptional factors and additional regulators is also critical for determining muscle cell fate and myotube/myofibers formation^2,5,6^.

MicroRNAs (miRNAs) modulate gene expression at the post-transcriptional level either by promoting mRNA degradation or inhibiting translation through complementary targeting 3’ untranslated regions (3’-UTRs) of specific mRNAs^2,6^. Many studies have demonstrated that miRNAs participate in skeletal muscle differentiation. The muscle-specific miRNAs, miR-206, miR-1, and miR-133, are abundantly expressed during skeletal muscle differentiation, and promote muscle differentiation by inhibition specific transcription repressors^7–10^. In addition, many non-muscle specific miRNAs also regulate muscle differentiation by post-transcriptional mechanisms that affect the presence and functions of the myogenic factors, either positively or negatively.

Our previous work focused on studying the biological roles of SETD3, which has been reported as a histone H3 Lys4 and Lys36 methyltransferase^11^. But very recent two studies clearly demonstrated that SETD3 is an actin-specific histidine methyltransferase^12,13^. We have shown that SETD3 is a cell-cycle regulated protein, and abnormal high level of SETD3 would lead to liver tumorigenesis^14^. A previous study has suggested that SETD3 is capable to interacting with MyoD and synergistically binding to the promoter of several muscle-related genes, thereby promoting muscle cell differentiation^11^. Knockdown of *SETD3* markedly impairs the differentiation processes, indicating its important role in muscle differentiation. However, how SETD3 is regulated during this process is completely unknown.

In this study, we hypothesized that *SETD3* gene is post-transcriptionally repressed by miRNAs. We uncovered that miR-15b and miR-322 could repress *SETD3* expression by targeting the 3′-UTR region in skeletal muscle cells. Furthermore, we revealed that two known transcription factors, E2F1 and FAM3B, could regulate miR-15b or miR-322 expression, respectively, during muscle cell differentiation. Thus, our results established a regulatory network between transcription factors, miRNAs, and an epigenetic modifier SETD3, which highlights a protein-microRNA involved cascade regulatory mechanism during skeletal muscle differentiation.

## Results

### SETD3 is required for C2C12 cell differentiation

Previous study suggested that SETD3 regulates muscle differentiation^11^. To confirm this, we first generated a monoclonal SETD3 antibody to detect endogenous SETD3 protein. This anti-SETD3 antibody specifically recognizes the SETD3 protein, as detected SETD3 signal was diminished when *SETD3* gene was knocked out in Hela S3 cells and overexpression of SETD3 constructs from either human or mouse species in the *SETD3* knockout cell line displayed specific bands (supplementary Fig. S1a). In addition, this anti-SETD3 antibody also recognizes endogenous SETD3 in C2C12 mouse myoblast cells, and knockdown of mouse *SETD3* by stable expression of two different sh*SETD3* constructs exhibited significant reduction of SETD3 level, indicating its specificity and species reactivity against mouse homolog SETD3 as well (supplementary Fig. S1a). Next, to examine whether SETD3 is required for cell differentiation, C2C12 cells was induced by cultured in the differentiation medium (DM), and expression of *SETD3* in both transcriptional levels and protein levels were examined. Consistent with previous results, transcription levels of several key regulatory factors including *MYF5, MYOG, TNNT2/Troponin*, and *MYH1/MYHC* were gradually increased during differentiation, with a similar trend of *SETD3* expression, indicating cell differentiation occurred (Fig. S1b)^2^. Intriguingly, we found that the protein levels of SETD3 displayed an increase at the early stage of differentiation, but showed a reduction when MHC protein was significantly accumulated, which may suggest a complicated regulatory mechanism of SETD3 involved in muscle differentiation (Fig. S1c). To rule out the possibility that the reduction of SETD3 protein level at the late stage of differentiation is due to our home-made antibody recognition issue, a commercial available antibody was utilized to examine SETD3 protein levels, and a similar expression pattern of SETD3 protein was observed (Fig. S1c). In addition, both antibodies were verified using two different synthesized siRNA oligos targeting *SETD3*, which confirmed the specificity of both antibodies (Fig. S1d). Consistent with previous report that knockdown of *SETD3* severely slows muscle cell differentiation based on the observation of cell morphology and differentiation gene expression^11^, we also observed knockdown of *SETD3* remarkably delayed cell differentiation, based on the divergence of cell morphology (Fig. S1e, f). Moreover, the protein levels of MHC as well as the mRNA levels of various differentiation markers were significantly reduced compared to the control cells during the progression (Fig. S1g, h). Therefore, our data support that SETD3 is required for C2C12 muscle cell differentiation.

### Identification of miRNAs that might affect *SETD3* expression

**Fig. 1:**
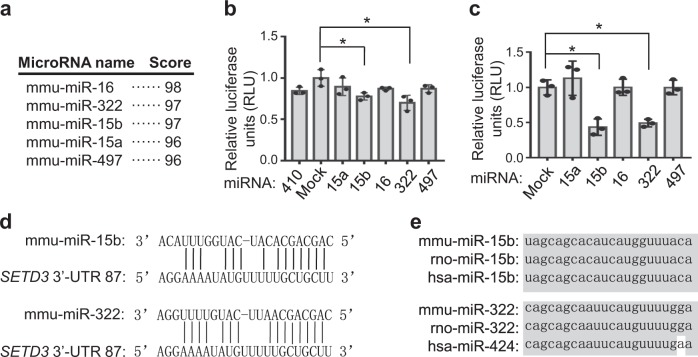
MiR-15b and miR-322 were identified as potential miRNAs targeting the 3′-UTR of *SETD3* gene. **a** List the top 5 of predicted miRNAs targeting the 3′-UTR of mouse *SETD3* gene. Score represents the potential capability of miRNA binding to 3′-UTR of *SETD3* gene. **b**, **c** Dual-luciferase reporter assays were performed using the indicated miRNAs co-transfected with a dual-luciferase reporter construct containing either a long 3′-UTR fragment (756 bp, panel **b**) or a short 3**′**-UTR fragment (23 bp, panel **c**) of *SETD3* gene. Dual-luciferase activities were normalized to the mock control, which was set as 1. Experiments were performed in three biological duplicates, and data are presented as mean ± SD. **d** Sequence alignment of mouse miR-15b or miR-322 with the 3′-UTR of *SETD3* gene was shown. **e** Homologous comparisons of miR-15b or miR-322 in different species. mmu Mus musculus, hsa Homo sapiens, rno Rattus norvegicus

We are interested in how *SETD3* levels are regulated at post-transcriptional levels during cell differentiation. Thus, we attempted to identify whether miRNAs might regulate expression of *SETD3*. To this end, the 3′ end of untranslated region (3′-UTR, nt 1786-2541) of mouse *SETD3* gene was selected for searching potential miRNAs using miRanda and TargetScan softwares^15,16^. Based on the predicted scores, we obtained several potential miRNAs and the top 5 candidates were selected (Fig. 1a). Interestingly, the binding regions of these five potential candidates are nearly identical, which are located from nt 1872 to nt 1894 in the 3′-UTR of *SETD3* gene. To identify which miRNAs might regulate *SETD3* expression, we first cloned the full-length (756 nt) 3′-UTR of the mouse *SETD3* gene and inserted into the downstream of a dual-luciferase reporter construct^17^. After the reporter construct was transfected into 293 T cells, we observed that only miR-15b or miR-322, but not other tested miRNAs, inhibited luciferase activity compared with the control construct (Fig. 1b). MiR-410 has been known to be not involved in regulation of *SETD3* expression, which served as a negative control. To further confirm this, a short 3′-UTR sequence (23 nt) that only contains the predicted binding sites shared by all the 5 miRNAs were inserted into the downstream of a dual-luciferase reporter construct. Again, we found that only miR-15b and miR-322 showed repressive effect towards luciferase activity (Fig. 1c). Of note, miR-15b and miR-322 share the same 3′-UTR region of *SETD3* gene, but with a slight difference in the seed region (Fig. 1d). Moreover, these two miRNAs are highly conserved among different species, suggesting their intrinsic function (Fig. 1e).

### MiR-15b and miR-322 directly targeted the 3′-UTR of *SETD3* gene

**Fig. 2:**
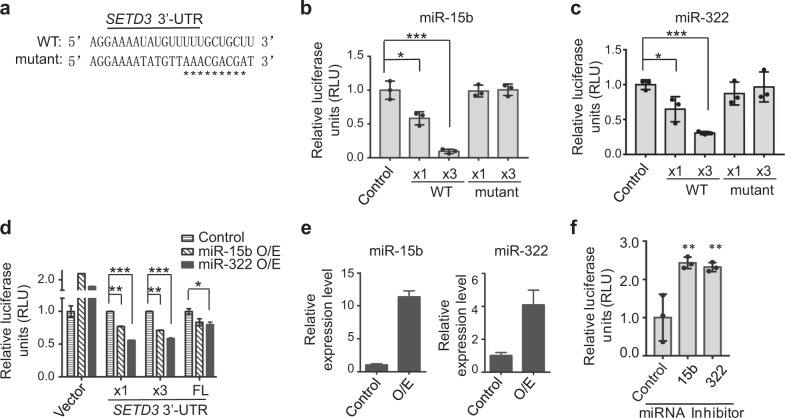
MiR-15b and miR-322 directly targeted the 3′-UTR of *SETD3* gene in vitro. **a** Sequence alignment of the short 3′-UTR fragment of the wild-type or the mutant *SETD3* was shown. The mutated sites in the seed region of the miRNAs were pointed out by “*“. **b**, **c** Luciferase reporter assays were performed using miR-15b (**b**) or miR-322 (**c**) co-transfected with a dual-luciferase reporter construct containing different lengths of 3′-UTR of *SETD3*. WT wild- type, x1 a single 3′-UTR sequence, x3 triple 3′-UTR repeat sequences. **d** The luciferase reporter constructs containing different 3′-UTR fragments of *SETD3* were transfected into C2C12 cells stably expressing pri-miR-15b or pri-miR-322 construct. Luciferase activities from the indicated cells were measured. FL the full length of the 3′-UTR fragment (756 bp). **e** Relative expression levels of miR-15b and miR-322 shown in **d** were measured by RT-qPCR. **f** Luciferase reporter assays were performed using the indicated miRNA inhibitors co-transfected with a dual-luciferase reporter construct containing short length of 3′-UTR of *SETD3*. The relative luciferase activities were quantified from three biological repeats

To validate whether miR-15b and miR-322 indeed targeted the 3′-UTR of *SETD3* gene, the predicted seed sequences of the short 3′-UTR in the luciferase reporter were mutated, and luciferase assays were performed as described above (Fig. 2a). We noticed that, when the reporter construct containing triple repeats of the short 3′-UTR of *SETD3*, the luciferase activity was repressed by both miR-15b and miR-322 more efficiently than the one containing a single copy of the short 3′-UTR of *SETD3*. In contrast, the luciferase activity remained invariable after the seed sequences in the 3′-UTR were mutated, compared to the control sample (Fig. 2b, c). When the indicated luciferase reporter constructs were transfected into cells that stably express pri-miR-15b or pri-miR-322, the luciferase activities were repressed by these miRNAs, but not by the empty luciferase reporter vector (Fig. 2d, e). In contrast, cotransfection of the indicated miRNA inhibitors with the luciferase reporter constructs into cells, the luciferase activities were enhanced compared to the control, suggesting their repressive roles of miRNAs in *SETD3* gene expression (Fig. 2f). Therefore, these results provided clear evidence showing that miR-15b or miR-322 can directly target the 3′-UTR of *SETD3* gene in vitro.

### MiR-15b and miR-322 repress *SETD3* expression through binding to the 3′-UTR of *SETD3*

**Fig. 3:**
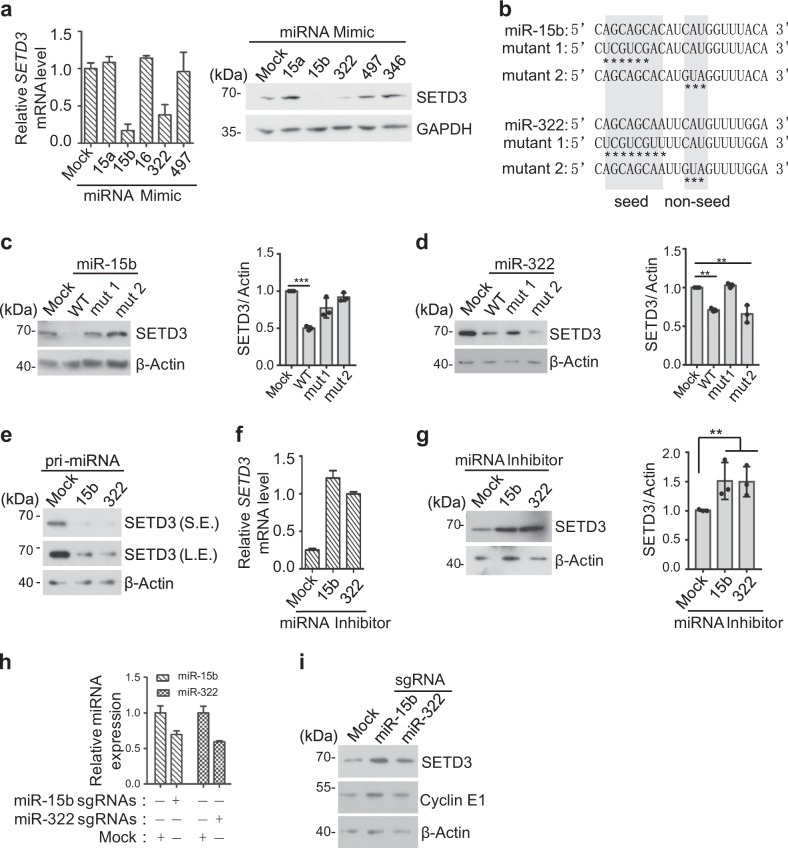
MiR-15b and miR-322 repress *SETD3* expression in vivo. **a** Transcriptional and protein levels of SETD3 in C2C12 cells expressing various miRNA mimics were examined by RT-qPCR (left panel) and western blot (right panel) analyses. **b** Sequences of the wild-type or mutants of miR-15b and miR-322 were shown. “*“ indicates the mutated sites. **c**, **d** SETD3 protein levels in cells expressing the indicated miRNA mimics or mutants shown in **b** were examined by western blot (left panel). The relative SETD3 levels normalized to β-Actin were quantified using ImageJ (right panel). **e** SETD3 protein levels in cells stably expressing the indicated pri-miRNAs were examined by western blot. **f**, **g** Transcriptional levels and protein levels of SETD3 in cells transfected the indicated miRNA inhibitors were examined by RT-qPCR (**f**) and western blot analysis (**g**, left panel). The relative SETD3 protein levels were quantified (**g**, right panel). **h**, **i** Transcriptional levels of miRNAs in cells transfected with sgRNA targeting miR-15b or miR-322 were examined by RT-qPCR in **h**, and protein levels of SETD3 were examined by western blot in **i**. The error bars in each quantification plot represent mean ± SD from three biological repeats

Next, we further explore whether miR-15b and miR-322 could repress *SETD3* expression in vivo. Five different miRNA sense oligos were transfected into C2C12 cells and SETD3 transcriptional and protein levels were examined by real-time quantitative PCR (RT-qPCR) and Western blot analyses. Consistently, only miR-15b or miR-322 remarkably reduced *SETD3* levels (Fig. 3a). Furthermore, we observed that transfection of the wild-type miRNA oligos, but not the miRNA-15b or miRNA-322 mutants in which either the seed sequences or the non-seed sequences have been changed, was able to affect SETD3 protein levels dramatically (Fig. 3b–d). The inert effect of miRNA-322 mutant 2 on *SETD3* might result from its non-essential role of targeting 3′-UTR of *SETD3* gene (Fig. 3d). As expected, overexpression of primary miR-15b or miR-322 construct in turn inhibited SETD3 protein levels (Fig. 3e). In addition, after antisense oligos of miR-15b or miR-322 (miRNA inhibitor) were transfected into C2C12 cells, both the transcriptional levels and the protein levels of SETD3 markedly increased compared to the control transfection (Fig. 3f, g). Alternatively, small guide RNA (sgRNA) was utilized to investigate the impact on *SETD3* expression. The two sgRNAs were able to decrease expression level of miR-15b or miR-322, respectively (Fig. 3h). Consistently, SETD3 protein levels were increased by sgRNA knockdown of miRNAs, suggesting *SETD3* may be the target of the two miRNAs in C2C12 cells (Fig. 3i). Consistent with previous reports that *CCNE1* was also targeted by miR-15b and miR-322, the encoded Cyclin E1 protein levels were moderately increased with knockdown of these two miRNAs^18–21^. Importantly, the effect of two sgRNAs on *SETD3* were not due to off-target effect, as coexpression of sgRNAs and their corresponding miRNA mimics compromised an accumulation of SETD3 caused by transfection sgRNAs alone (supplementary Fig. S2). Taken together, we conclude that miR-15b and miR-322 can directly target the 3′-UTR of *SETD3* gene, which lead to inhibition of *SETD3* expression.

### MiR-15b and miR-322 repress myoblast differentiation

**Fig. 4:**
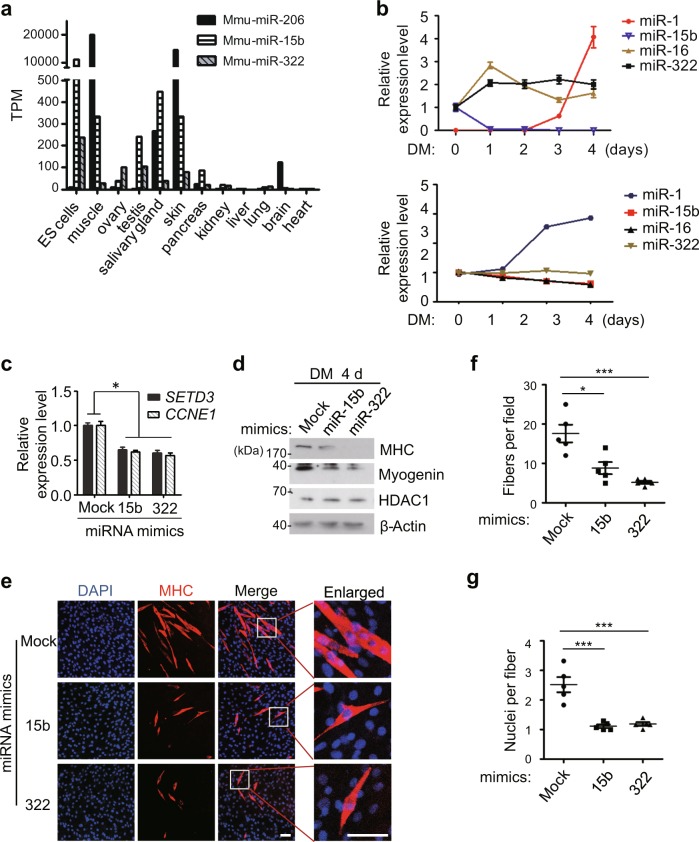
Overexpression of miR-15b or miR-322 delayed myoblast differentiation. **a** The expression levels of the indicated miRNAs in embryonic stem (ES) cells and various mouse tissues were plotted using the datasets in GEO. **b** The expression levels of the indicated miRNAs during C2C12 cells differentiation were analysed by RT-qPCR (top panel) or by using the datasets in GEO (bottom panel). **c**–**g** C2C12 cells were transfected with the indicated miRNA mimics, and induced differentiation for 4 days. **c** The mRNA levels of *SETD3* and *CCNE1* from differentiated cells were examined by RT-qPCR. **d** The differentiation protein markers in the transfected cells were analyzed by western blot analysis. **e** Immunofluorescence staining was performed using a specific antibody against MHC (red). DAPI stains nuclei. Scale bar: 100 μm. **f** MHC-positive cell numbers in certain field of the indicated C2C12 cells describe in **e** were quantified. **g** Quantitative analyses of nuclei number per fiber from the indicated C2C12 cells. Data represent the mean ± SD from three independent experiments

Since both miR-15b and miR-322 can repress *SETD3* expression, we want to determine how these two miRNAs are expressed during muscle cell differentiation. To do this, we first utilized a database published previously and analyzed the expression levels of these miRNAs in various mouse tissues^22^. Consistently, the expression level of miR-206, a muscle-specific miRNA, is enhanced over 2000 fold in mouse muscle compared with that in mouse embryonic stem (ES) cells^10^. Interestingly, the expression levels of miR-15b and miR-322 are significantly decreased in mouse muscle compared to those in ES cells (33-fold or 8-fold reduction respectively) (Fig. 4a and supplementary Table S1). These two miRNAs are comparably expressed in other tissues, suggesting that they are not muscle-specific miRNAs. To this end, we then investigated the dynamic expression levels of miR-15b and miR-322 during C2C12 differentiation by RT-qPCR. MiR-1 was served as a positive control, as its expression level has been reported to gradually increase during muscle differentiation^8^; whereas miR-16 was served as a negative control, as it does not target *SETD3* demonstrated in our result (Fig. 1b, c). We observed that miR-15b levels were declined during C2C12 differentiation, which is inversely correlated with *SETD3* levels. Intriguingly, miR-322 levels remained unchanged, which is consistent with the results that we analyzed using the public GEO database (NCBI, Gene Expression Omnibus, www.ncbi.nih.gov/geo) (Fig. 4b). Next, undifferentiated C2C12 cells were transfected with synthetic miRNAs mimics, and treated with differentiation medium to induce myogenic differentiation for 4 days. RT-qPCR analysis showed that endogenous *SETD3* mRNA levels were decreased (Fig. 4c). Western blot analysis confirmed that the protein levels of differentiation markers, such as MHC and Myogenin, decreased upon transfection of these two miRNAs mimics (Fig. 4d). Furthermore, immunofluorescence assays showed that myoblasts transfected with those miRNA mimics attenuated myoblast differentiation, as visualized by a significant decrease in the number and size of myotubes (Fig. 4e, f). Of note, the nuclei numbers per fiber were dramatically decreased, illustrating the defect of myoblast fusion into myotubes (Fig. 4g). Thus, we concluded that miR-15b and miR-322 may function in repression of myoblast differentiation.

### MiR-15b and miR-322 inhibit *SETD3* to regulate muscle cell differentiation

**Fig. 5:**
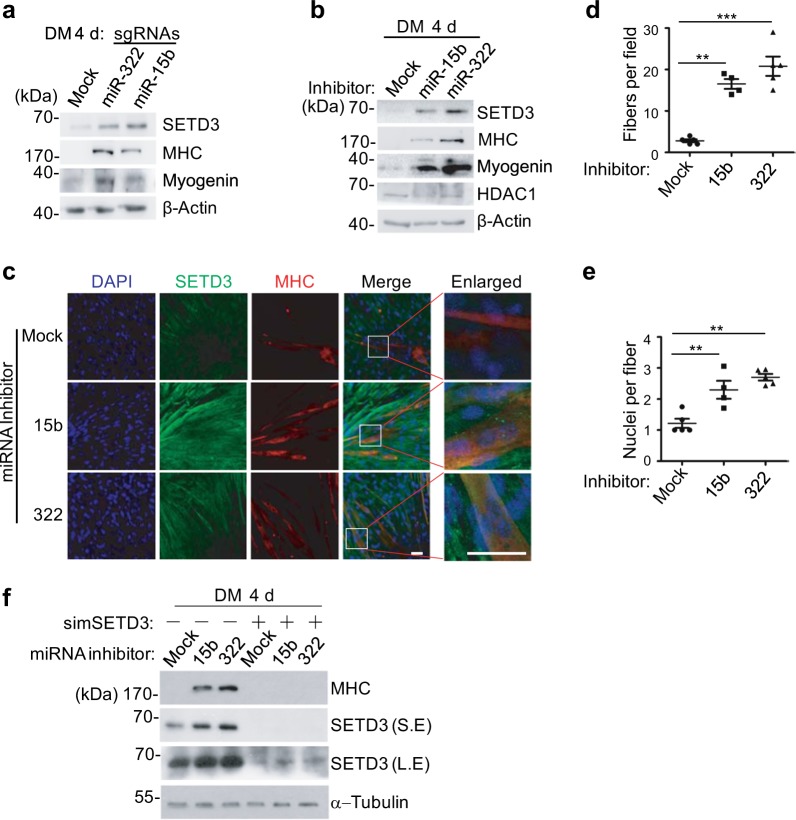
MiR-15b and miR-322 inhibit SETD3 to regulate muscle cell differentiation. **a**, **b** C2C12 cells transfected with the indicated sgRNAs (**a**) or miRNA inhibitors (**b**) targeting miR-15b or miR-322 were differentiated for 4 days, and the levels of the indicated proteins were examined by western blot. **c** Immunofluorescence analyses of the indicated differentiated C2C12 cells were stained with antibodies against SETD3 (green) and MHC (red). DAPI stains nuclei. Scale bar: 100 μm. **d** MHC-positive cell numbers in certain field of the indicated C2C12 cells describe in **c** were quantified. **e** Quantitative analyses of nuclei number per fiber from the indicated C2C12 cells. Data represent the mean ± SD from five fields. **f** Knockdown of *SETD3* rescues miR-15b or miR-322 mediated myoblast differentiation. The protein levels of MHC and SETD3 were examined by western blot. S.E. short exposure, L.E. long exposure

Next we determine whether reduction of miR-15b or miR-322 could derepress its negative role in muscle differentiation. Therefore, a pair of sgRNAs or miRNA inhibitors that targeted miR-15b or miR-322, respectively, was transfected into C2C12 cells. After 4 days induction of myogenic differentiation, a significant increase of SETD3 levels accompanied with elevated levels of myogenic markers MHC and Myogenin were observed (Fig. 5a, b). Furthermore, immunofluorescence assays showed that myoblasts transfected with miRNA inhibitors formed more myotubes (Fig. 5c). Quantitative measurement of the numbers of myotubes and the nuclei numbers per fiber demonstrated that repression of miR-15b or miR-322 promotes myoblast differentiation (Fig. 5d, e).

To validate whether miR-15b or miR-322 affects myoblast differentiation via regulating *SETD3*, a rescue experiment was performed. C2C12 cells were transfected with siRNA targeting *SETD3*. After 4 h treatment, the inhibitor of miR-15b or miR-322 was separately added into cells for additional 6 h. After continued to culture in fresh media for 24 h, cells were induced to differentiation for additional 4 days. Inhibition of endogenous miR-15b or miR-322 increased *SETD3* levels and facilitated cell differentiation; whereas knockdown of endogenous *SETD3* rescued miR-15b or miR-322 inhibitors-mediated cell differentiation, evaluated by MHC levels (Fig. 5f). This result suggested that miR-15b and miR-322 repress muscle cell differentiation via inhibition of *SETD3* expression.

### E2F1 and FAM3B regulate *SETD3* levels through controlling miR-15b or miR-322 expression, respectively

**Fig. 6:**
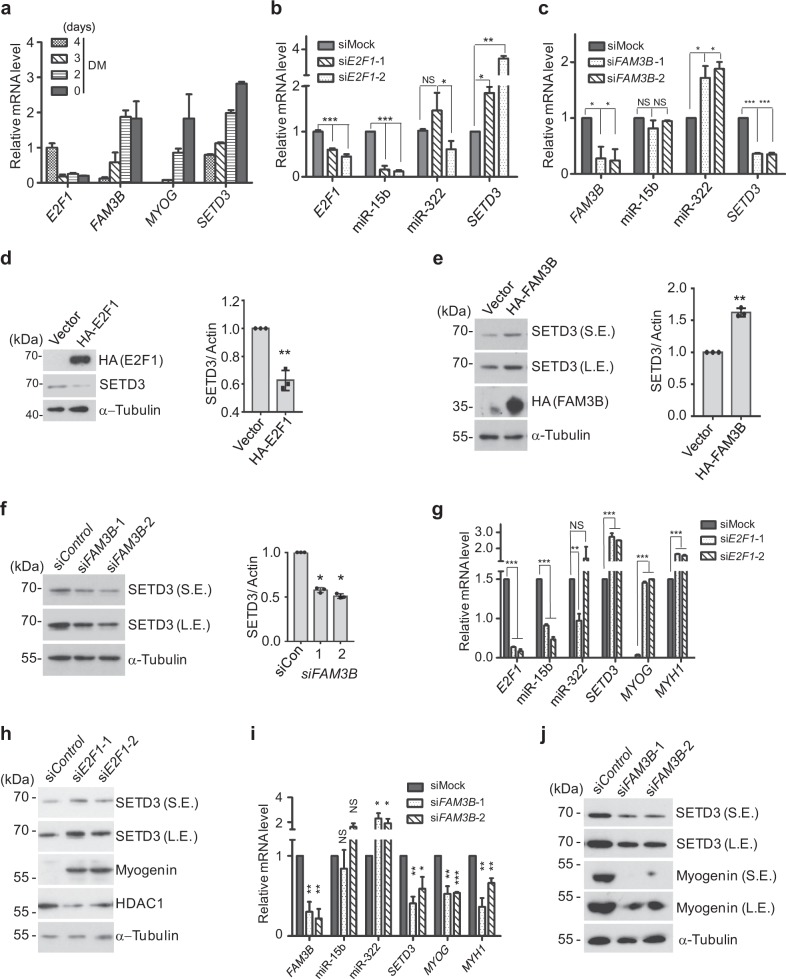
E2F1 and FAM3B regulate SETD3 levels through controlling miR-15b or miR-322 expression. **a** The transcriptional levels of the indicated genes during muscle differentiation were examined by RT-qPCR. **b**, **c** The transcriptional levels of miR-15b or miR-322 after knockdown *E2F1* (**b**) or knockdown *FAM3B* (**c**) were examined. The relative SETD3 levels were shown. **d**, **e** Western blot assays were performed to examine SETD3 levels after overexpression of E2F1 (**d**, left panel) or FAM3B (**e**, left panel) in C2C12 cells. Data represent the mean ± SD from three independent experiments (right panel). **f** Knockdown of FAM3B by two different siRNAs reduces SETD3 levels. **g**, **i** C2C12 cells were transfected with siRNAs targeting E2F1 or FAM3B, respectively, and induced to differentiate for 3 days. The indicated miRNAs or mRNA levels were examined by RT-qPCR. Data represent the mean ± SD from three independent experiments. **h**, **j** The same set of cells described in panel **g**, **i** were subjected to immunoblotting against the indicated antibodies. S.E. short exposure, L.E. long exposure

Next, we want to address how expressions of miR-15b and miR-322 are regulated during muscle differentiation. Previous studies have demonstrated that the pivotal transcription factor E2F1 directly targets promoters of miR-15 and miR-16 clusters and E2F1 inhibits myogenic differentiation^23,24^. Meanwhile, Zhang et al. recently reported that FAM3B inhibits miR-322 expression during high glucose induced vascular smooth muscle cell proliferation^25^. These results prompt us to investigate whether E2F1 and FAM3B regulate expression of miR-15b and miR-322 during skeletal muscle differentiation, respectively. To test this, we first examined the expression profiles of *E2F1* and *FAM3B*. As expected, during myogenic differentiation process, *E2F1* expression was reduced, whereas *FAM3B* expression was gradually increased (Fig. 6a). Consistently, knockdown of the positive transcriptional regulator E2F1 remarkably repressed miR-15b expression, and consequently increased *SETD3* expression, but had no obvious impact on miR-322 expression (Fig. 6b). Meanwhile, knockdown of the negative regulator FAM3B promoted miR-322 expression, and consequently reduced *SETD3* expression, but had no effect on miR-15b expression (Fig. 6c).

We speculate that E2F1 and FAM3B could regulate muscle differentiation by affecting *SETD3* levels. Thus, SETD3 levels were examined in C2C12 cells transfected with HA-tagged E2F1 by immunoblotting. Interestingly, overexpression of E2F1 reduced SETD3 protein level (Fig. 6d). Similarly, when we altered FAM3B levels by overexpression or siRNA knockdown FAM3B, SETD3 protein levels were accordingly changed as expected (Fig. 6e, f). Moreover, if E2F1 was inhibited by siRNA knockdown, we observed much faster cell differentiation compared to the control cells after switching cells to the differentiation medium (Fig. 6g, h). In contrast, knockdown of FAM3B slowed down cell differentiation compared to the control cells, as both SETD3 and Myogenin levels were obviously decreased in same differentiation conditions (Fig. 6i, j). Together, these data indicated that E2F1 or FAM3B either positively or negatively regulates miRNAs, consequently affects SETD3 and muscle differentiation.

## Discussion

**Fig. 7:**
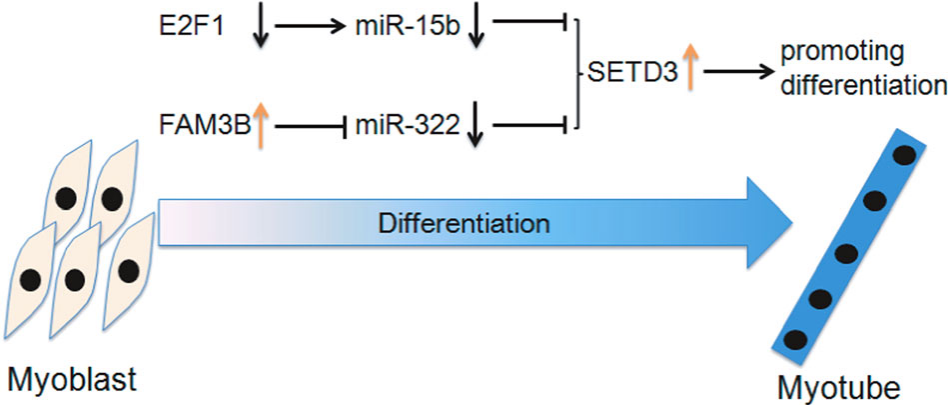
**A proposed model shows that SETD3 is involved in two parallel cascade pathways during muscle cell differentiation**

In this study, we uncover a novel function of miR-15b and miR-322 in C2C12 differentiation beyond their roles in cancers^26,27^. Furthermore, we verify that a well-known transcription factor E2F1 is required for the reduction of miR-15b expression and the upregulation of SETD3, thereby promoting myoblast transition to myotube formation. Meanwhile, we also illustrate that a negative transcription regulator FAM3B is upregulated during this differentiation process, accompanied with decreased miR-322 level as well as increased SETD3 level (Fig. 7). These two parallel pathways of regulation of SETD3 expression highlight the importance of a protein-miRNAs interplay network during skeletal muscle differentiation.

MiRNAs have been elucidated to participate in almost every aspect of biology. For instance, the miR-15a/16-1 and miR-15b/16-2 clusters have been shown to regulate cell cycle and apoptosis by targeting *CCND3* or *CCNE1*^24^. MiR-15b has been shown to play roles in adipogenesis, lipid metabolism, and modulating DNA damage response^28–30^. In addition, miR-15a and miR-15b also functions as tumor suppressors, especially in B-cell oncogenesis, suggesting their potential clinical application^26,31^. It is worthy to note here that we uncovered miR-15b, but not miR-15a, regulates skeletal muscle differentiation, although these two miRNAs share very similar sequences. We have provided clear evidence showing that the unique role of miR-15b in muscle differentiation (Figs. 1c and 3c). Given that miR-15a and miR-15b are located at different chromosome loci, it is conceivable that these two miRNAs have distinct roles^32^.

Unlike miR-15b, miR-322 has been indicated to regulate muscle differentiation as well as cardiomyocyte specification^33,34^. As an X-chromosome miRNA, miR-322/-503 cluster specifically drives a cardiomyocyte program meanwhile inhibiting neural lineages^33^. MiR-322 can promote osteoblast differentiation by downregulation of Tob2 and Tob2-regulated osteogenic genes^34^. Meanwhile, miR-322 represses muscle differentiation, as overexpression of miR-322 mimics dampened myotube formation but promoted bone formation^34^. Consistently, we show here that inhibition of miR-322 significantly accelerate myotube formation, further confirming its negative role in myoblast differentiation (Fig. 5). In contrast, miR-322 can promote cell cycle quiescence and differentiation by down-regulation of Cdc25A^27^. Despite of this, whether miR-322 represses muscle differentiation has not been examined in that study. Our data have demonstrated that miR-322 indeed represses muscle differentiation. Nevertheless, why constant transcript levels of miR-322 are sustained during myoblast differentiation awaits further investigation.

It is interesting to understand how expression of the two miRNAs themselves is regulated during muscle differentiation. Here we provide evidence showing that E2F1 or FAM3B regulates expression of miR-15b or miR-322, respectively, during this process: (1) expression of E2F1 and FAM3B are dynamically altered from myoblast state to myotube formation; (2) E2F1 and FAM3B specifically control expression of the two miRNAs through directly targeting their corresponding promoters, respectively; (3) knockdown of *FAM3B* in C2C12 cells results in decreased *SETD3* expression, which are correlated with repression of muscle cell differentiation (Fig. 6). Actually, previous studies have shown many clues to support our findings. First, E2F1-mediated transcription plays an essential role in muscle differentiation and myogenesis^23,35^. E2F1 expression is irreversibly downregulated during C2C12 myoblast differentiation, whereas overexpression of E2F1 promotes myoblast proliferation and represses myogenic differentiation^23,36^. Second, E2F family members, including E2F1 and E2F3, can directly bind to the promoter of miR-15b-16-2, and positively regulate miRNA expression during cell proliferation^24,37^. In addition, FAM3B protein is significantly increased during the proliferation and migration of vascular smooth muscle cells, accompanied with the inhibition of miR-322-5p, linking FAM3B to miR-322 regulation^25^. Moreover, luciferase reporter assay has been shown that FAM3B represses transcription of miR-322 by binding the promoter of miR-322^25^. Therefore, we at the first time demonstrate that E2F1 and FAM3B can regulate SETD3 through two parallel miRNA regulatory pathways, and decipher a complex network during myogenic differentiation.

Using cultured myoblast cell system, our current studies convincingly demonstrate the function and regulation of miR-15b and miR-322 in myoblast differentiation. It will be important to determine whether the repression of *SETD3* by miRNAs contributes to skeletal muscle development and function. It will also be interesting to determine if miR-15b/miR-322 and SETD3 participate in skeletal muscle degeneration/regeneration process as well as human muscular diseases, such as rhabdomyosarcoma.

## Methods and Materials

### MiRNA prediction

MiRNAs potentially targeting the 3′-UTR of *SETD3* gene were predicted by the TargetScan (http://www.targetscan.org/mmu_71/) and the Miranda (http://34.236.212.39/microrna/home.do) websites.

### Construction of plasmids

The E2F1 and FAM3B from human cDNA library were transferred to pCS2-based Gateway vector containing 3xHA tag via LR reaction as described previously^38^.

### Cell culture and transfection

C2C12 mouse myoblasts were cultured in growth medium (GM) — DMEM containing 20% fetal bovine serum (FBS) and maintained in a humidified incubator with 5% CO_2_ at 37 °C. For myogenic differentiation, when confluence was reached to 80–90%, C2C12 cells were shifted into a differentiation medium — DMEM containing 2% horse serum (HS). 293 T cells were cultured in DMEM containing 10% FBS and maintained in a humidified incubator with 5% CO_2_ at 37 °C. For miRNAs and plasmids transfection, when cells reached 60–70% confluence, the miRNAs or plasmids were transfected by the transfection reagent MAX according to the manufacturer’s protocol. Cells were harvested in 36–48 h after transfection of plasmids or 48–96 h after transfection of siRNAs or miRNAs. Unless stated, 293T cells were only used for the luciferase reporter assays; C2C12 cells were mainly used for cell differentiation experiments.

The synthesized miRNA or siRNA sequences (GenePharma Com. from Shanghai) are below: miR-15b mimics (WT): 5′- cagcagcacauaucagguuuaca-3′; miR-15b mutant 1: 5′-cucgucgacaucaugguuuaca-3′; miR-15b mutant 2: 5′-cagcagcacauguagguuuaca-3′; miR-322 mimics (WT): 5′-cagcagcaauucauguuuugga-3′; miR-322 mutant 1: 5′-cucgucguuuucauguuuugga-3′; miR-322 mutant 2: 5′-cagcagcaauuguaguuuugga-3′; miR-15b inhibitor: 5′-UGAA- CCAUGAUGUGCUGCUA-3′; miR-322 inhibitor: 5′-UCCAAAACAUGAAUUGCUGCUG-3′; si-mE2F1-1: 5′-ATCTGACCACCAAACGCTT-3′; si-mE2F1-2: 5′-GCCCTTGACTATCACTTTGGT-3′; si-mFAM3B-1: 5′-CAAACTGAAGGCTCAAGCAAA-3′; si-mFAM3B-2: 5′-GCACTCTCTACAACATCGAA-3′.

### Western blot

Cells were lysed by RIPA buffer and added the bromophenol blue loading buffer, and then the samples were boiled for 10 min and centrifuged at 12,000 rpm for 5 min. The whole-cell lysate was separated into 8% SDS-acrylamide gels and transferred to PVDF membranes. After that, the membranes was blocks by 5% milk in TBST and probed with primary antibodies including mouse SETD3 (3B3, generated by Wuhan Dia-An Company), rabbit polyclonal SETD3 (Abclonal, A8071), MyoD1 (Proteintech, 18943-l-AP), HDAC1 (Abclonal, A2238), Cyclin E1 (Cell Signaling Technology, 20808 S), Myogenin (Abcam, ab124800; or Santa Cruz, D-10, sc-13137), MHC (Developmental Studies Hybridoma Bank, MF-20), β-Actin (Proteintech, 6008-I-Ig), and α-Tubulin (Sigma, T9026). For generation of mouse monoclonal SETD3 antibody, His-tagged full-length human SETD3 protein was expressed in *E. Coli* and purified as described previously^14^. Purified His-SETD3 proteins were immunizated into 5-8 weeks old Balb/C mice and boosted additional 4 times. After several steps including hybridoma production, screening, cloning, and expanding the hybridomas, a subclone named 3B3 was validated and amplified followed the procedure described as before^39^. Membranes were further probed with horseradish peroxidase (HRP)-conjugated secondary antibodies and the protein bands were visualized using chemiluminescence detection reagents.

### RNA extraction, reverse transcription, and real-time quantitative PCR

Total RNA was isolated from C2C12 cells with TRIzol (Life technologies). The mRNA reverse transcription and real-time PCR were according to the manufacturer’s protocol (TIANGEN). The miRNA reverse transcription and real-time PCR were using the Hairpin-it^TM^ Real-Time PCR Kit (Shanghai GenePharma). The primer sequences used in RT-qPCR are available upon request.

### Dual-luciferase reporter assays

The dual-luciferase reporter plasmid psiCHECK2 was generously gifted from Xiang-Dong Fu laboratory. The longer 3′-UTR fragment of *SETD3* gene was amplified by PCR from cDNA of C2C12 cells and cloned into psiCHECK2 vector’s downstream of the stop codon of Renilla luciferase gene. For Luciferase reporter assays, 20 nM miRNA and 10 ng plasmid were transfected into 293 T cells or C2C12 cells. After 24–48 h, cells were lysed and the luciferase activity was tested according to the manufacturer’s instructions (Promega).

### Generation of *SETD3* knockdown cell line

Short hairpin RNA fragments (shRNAs) of *SETD3* containing 5′- CATCACCATGTTCCTTGTTAA-3′ (sh*SETD3*-1) or 5′-GCTGGAGATCA- GATTTACATT-3′ (sh*SETD3*-2) were cloned into plko.1 vector using the restriction enzymes *EcoR*I and *Age*I (New England Biolabs). To obtain lentivirus, the knockdown plasmids were transfected into 293 T cells along with the helper plasmids pMD2G and psPAX2 using the ratio of 2:1:1. Cell culture medium was changed after 12 h transfection and virus were harvested 24 h later with filter. Cells were seeded into a 12-well plate 1 day before lentivirus infection. *SETD3* knockdown cells will be harvested after 36–48 h.

### Knockdown of miRNAs by CRISPR-Cas9 technology

We designed two sgRNAs each miRNA by the CRISPR Design Tool (http://tools.genome-engineering.org) and inserted them into pSpCas9 (BB)-2A-Puro vector. The sgRNA sequences are below: miR-15b-sgRNA-1: 5′-AGTACTGTAGCAGCACATCA-3′; miR-15b-sgRNA-2: 5′-CAAACATAATACAACTGTGA-3′; miR-322-sgRNA-1: 5′-CCCTTCGGAGTCAACGAGGG-3′; miR-322-sgRNA-2: 5′-GCGCTGCAACACCCCTTCGT-3′. After CRISPR-Cas9 plasmids transfected and selected by puromycin for 2-3 days, C2C12 cells were harvested and *SETD3* expression level was analyzed by western blot.

### Pri-miRNA overexpression system

Pri-miRNA sequences were searched from UCSC Genome Browser (http://genome.ucsc.edu) and a nucleotide segment containing mi-15b or mi-322 was cloned into pHAGE-CMV vector using the restriction enzymes *Not*I and *Xho*I (New England Biolabs). The primers used for construction of pri-miRNA are as follows: pri-miR-15b forward (F): 5′-ATAAGAATGCGGCCGCGCCACCGGCATTG-ACTTAGACCATAATC-3′; pri-miR-15b reverse (R): 5′-CCGCTCGAGCACTACGCCAATATTTACGTG- 3′; pri-miR-322 forward (F): 5′-ATAAGAATGCGGCCGCGCCACCCTGAGGTAAGAGTCTCCTCC-3′; pri-miR-322 reverse (R): 5′-CCGCTCGAGGTGACCCTCACTAGACTAA-G-3′. 293 T cells were infected and selected according to the lentiviral expression and packaging protocol described above. The packaged virus was used to infect C2C12 cells to generate pri-miRNA stably expressed cell lines.

### Immunofluorescence staining

C2C12 cells were cultured on glass coverslips, induced to differentiation for 4 days, fixed with 4% paraformaldehyde for 10 min, permeabilized with 0.1% Triton X-100 (Sigma) for 10 min, blocked with 3% BSA solution, incubated with an primary antibody (for MHC: 1:50; for SETD3 1:100) at 4 °C overnight, incubated with a secondary antibody at room temperature for 1 h. The coverslips were stained with DAPI and mounted. Immunofluorescence images were captured under a confocal laser-scanning microscope (Leica SP8).

### Statistical analysis

For quantification of the western blot data, ImageJ software was used to measure the relative intensity of each band. Data are presented as mean ± standard deviation (SD) from at least three biological replicates, and the difference between any two groups were compared by Student’s *t-*test using Prism 5 software. NS not significance, *p < 0.05, **p < 0.01, ***p < 0.001.

## Supplementary information

supplemental materials
