# Supplementary material for: MiR-15b and miR-322 inhibit SETD3 expression to repress muscle cell differentiation
Source: Cell Death Dis. 2019 Feb 22;10(3):183. doi: 10.1038/s41419-019-1432-5 (PMC6385263; doi:10.1038/s41419-019-1432-5)
Supplement: Supplementary file 1 — supplemental materials [file 41419_2019_1432_MOESM1_ESM.pdf]

Supplemental Materials

1. Table S1. Expression levels of miRNAs in various tissues.

2. Fig. S1 SETD3 is required for C2C12 cell differentiation.

3. Fig. S2 Exclusion the off-target effects of sgRNAs on SETD3.

Table S1. Relative expression levels of miRNAs in various tissues[a]

Tissues	miR-15b	miR-322	miR-206-3p[b]
embryonic stem cells	10943.77	237.7131	10.57885424
muscle	331.4213155	28.25951529	20123.60084
ovary	39.41509085	102.5872228	9.718789525
testis	240.7160123	104.7560424	2.228851966
salivary gland	445.4428612	39.27338451	265.4053985
skin	331.1853769	80.42207444	14464.45567
pancreas	85.41122837	20.09675962	24.17891391
kidney	19.38921237	15.36503622	3.841259055
liver	1.161430069	0.193571678	0.774286713
lung	10.84149403	14.16388737	0.699451228
brain	6.480972405	3.780567236	124.5786918
heart	1.55497719	2.526837934	3.109954381

[a]Data was obtained from miRbase (a public GEO database, www.ncbi.nih.gov/geo).
[b]The biogenesis of miR-206 is unique in that the primary mature transcript is generated from the 3p arm of the precursor hairpin rather than the 5p arm.

Zhao et al. Figure S1

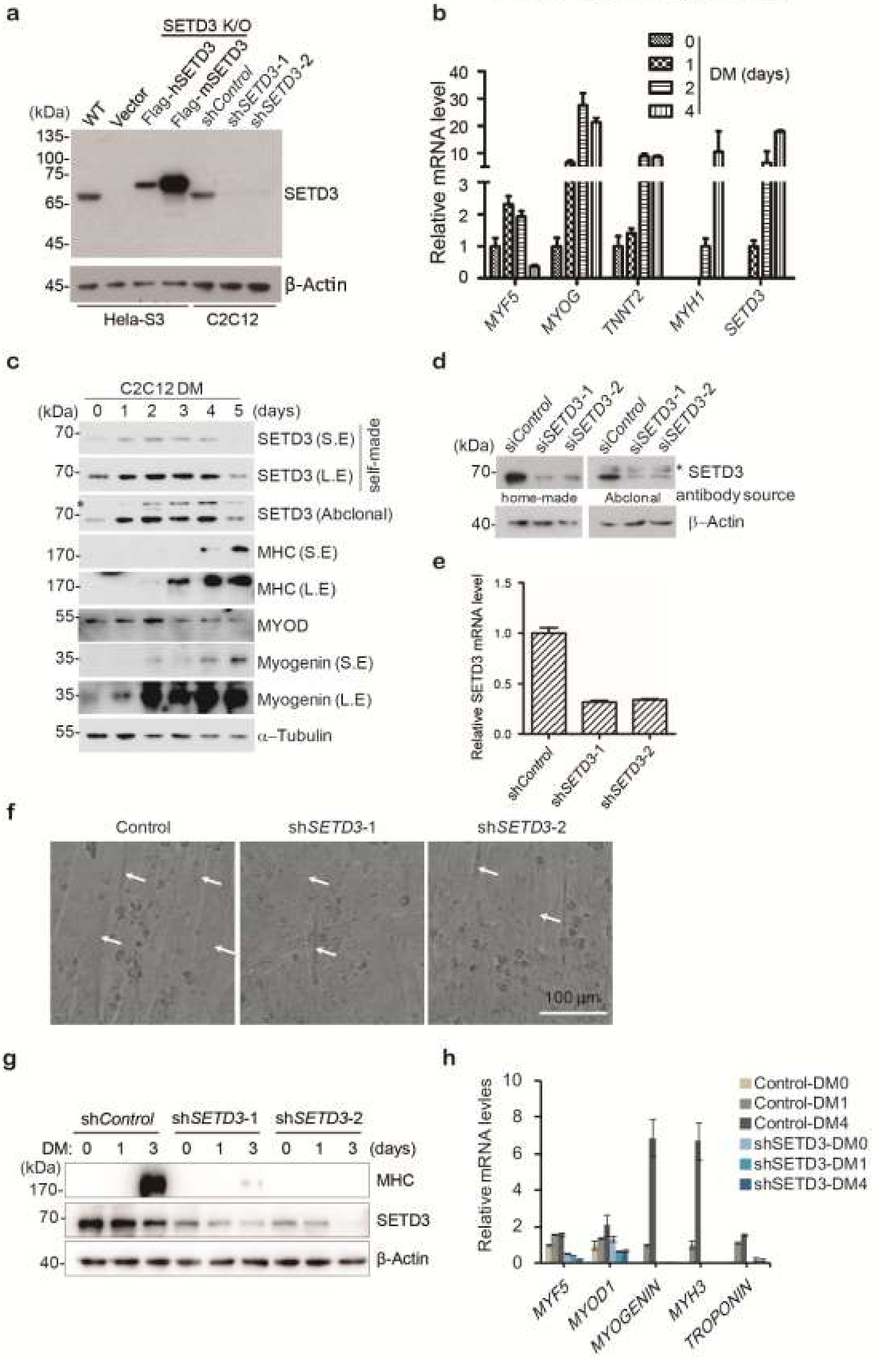

Fig. S1 SETD3 is required for C2C12 cell differentiation. a Validation of the monoclonal SETD3 antibody. Indicated cell lysates from wild-type Hela S3 cells, *SETD3* knockout Hela S3 cells transfected either vector, Flag-tagged human SETD3, or Flag-tagged mouse SETD3 constructs, or C2C12 cells transfected with shRNA control or shRNAs targeting mouse SETD3 (sh*SETD3*-1, sh*SETD3*-2) were subjected to Western blot analysis probing with the purified mouse SETD3 antibody. **b** and **c** C2C12 cells were cultured in the differentiation medium (DM) for the indicated times. Transcriptional levels and protein levels of SETD3 were examined by RT-qPCR (panel **b**) and Western blot (panel **c**) analyses. SETD3 protein levels were examined by our home-made antibody and a commercial antibody purchased from Abclonal Company. S.E.: short exposure; L.E.: long exposure. Asterisk represents a non-specific band. **d** *SETD3* knockdown efficiency by two different siRNA oligos targeting *SETD3* was examined using Western blot analysis, and two different sources of antibodies described above were compared. Asterisk represents non-specific bands. **e** *SETD3* knockdown efficiency by transfecting two different short hairpin RNAs (shRNAs) targeting *SETD3* was measured using RT-qPCR assays. **f** Representative phase-contrast images of cell morphology were taken in the indicated C2C12 cells after 4 days grown in DM. Arrows represent elongated myotubes. *Scale bar*: 100 μm. **g** C2C12 cells transfected with the indicated shRNA constructs were cultured in DM for the indicated days. SETD3 protein levels were examined by Western blotting. MHC is used as markers indicating the late stage of cell differentiation. β-actin served as a loading control. **h** Relative mRNA levels of several muscle differentiation markers were measured in shControl or sh*SETD3* cells taken at the indicated differentiated days by RT-qPCR. DM0: grown in differentiation medium for 0 day; DM1: grown in differentiation medium for 1 day; DM4: grown in differentiation medium for 4 days.

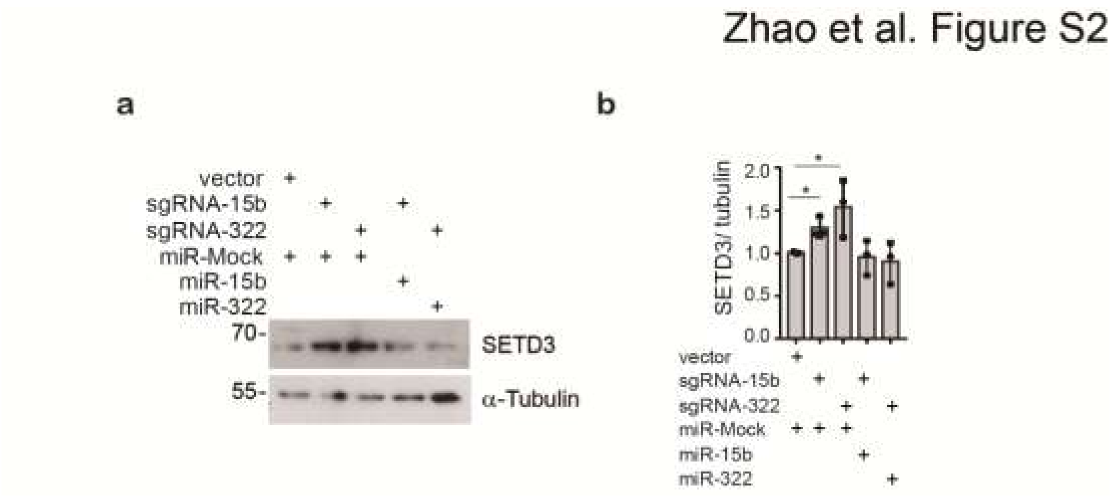

Fig. S2 Exclusion the off-target effects of sgRNAs on SETD3. a C2C12 cells that were either transfected sgRNA targeting miR-15b or miR-322 alone or co-transfected sgRNA with the corresponding miRNA mimics together were subjected to SDS-PAGE following Western blot analysis, SETD3 protein levels were examined. **b** Relative SETD3 protein levels normalized to α-tubulin were plotted and quantified from three independent experiments. The error bars represent mean±SD from three independent experiments.
